# Supplementary material for: Liver Transplantation for Unresectable Colorectal Liver Metastases: A Scoping Review on Redefining Boundaries in Transplant Oncology
Source: Curr Oncol. 2025 Aug 28;32(9):481. doi: 10.3390/curroncol32090481 (PMC12468046; doi:10.3390/curroncol32090481)
Supplement: Supplementary file 1 [file curroncol-32-00481-s001.zip › curroncol-3750230-supplementary.pdf]

Current Oncology

MDPI

Table S1. PRISMA-ScR Checklist+G10:J24.

Section	Item	Checklist Item Description	Reported in Manuscript
**TITLE**	1	Identify the report as a scoping review.	Title page – "Scoping Review" clearly stated
**ABSTRACT**	2	Provide a structured summary including background, objectives, eligibility criteria, information sources, charting methods, results, and conclusions.	Abstract – structured with all required elements
**INTRODUCTION**	3	Describe the rationale for the review in the context of what is already known.	Introduction, paragraphs 1–3
	4	State the objectives or questions the review addresses.	Introduction, final paragraph
**METHODS**	5	Specify inclusion and exclusion criteria.	Methods, paragraph 2
	6	Describe all information sources (databases, registers, websites, etc.) with dates of coverage.	Methods, paragraph 3
	7	Present the full electronic search strategy for at least one database, including any limits used, so it can be repeated.	Appendix/Table S1
	8	Describe the process for selecting sources of evidence (screening and eligibility).	Methods, paragraph 3
	9	Describe the method(s) of charting data from the included sources of evidence.	Methods, paragraph 4
	10	List and define all variables for which data were sought.	Methods, paragraph 4
	11	If done, describe methods used for critical appraisal of individual sources of evidence.	Not applicable – critical appraisal not performed
	12	Describe how the data were synthesized.	Methods, paragraph 5
**RESULTS**	13	Provide numbers of sources screened, assessed for eligibility, and included in the review, with reasons for exclusions.	Results, first paragraph + PRISMA flow diagram (Figure 1)
	14	Present characteristics for each source of evidence.	Results, Table 1
	15	If done, present data on critical appraisal of included sources.	Not applicable
	16	Present results for each included source of evidence.	Results, subsections summarizing each study
	17	Summarize and/or present results in relation to the review questions and objectives.	Results, final subsection
**DISCUSSION**	18	Summarize the main results (including an overview of concepts, themes, and types of evidence available).	Discussion, first 2 paragraphs
	19	Discuss limitations of the evidence included in the review.	Discussion, penultimate paragraph
	20	Provide a general interpretation of the results with implications for future research, policy, or practice.	Conclusions section
**FUNDING**	21	Describe sources of funding for the included sources of evidence and for the review itself.	Funding statement in the manuscript
**CONFLICTS**	22	Declare competing interests.	Conflicts of interest statement in the manuscript

**Supplementary Table S2. PRISMA Flow Diagram (Text Version)**	
**Identification**	**Records identified through database searching: n = 450**
	**Additional records identified through other sources: n = 45**
	**Total records identified: 495**
**Screening**	**Records after duplicates removed: n = 380**
	**Records screened: n = 380**
	**Records excluded: n = 280**
**Eligibility**	**Full-text articles assessed for eligibility: n = 100**
	**Full-text articles excluded, with reasons: n = 60**
	**- Not colorectal liver metastases**
	**- Wrong study design (e.g., case reports, editorials)**
	**- Incomplete outcome data**
**Included**	**Studies included in qualitative synthesis: n = 10**
